# Cellpose 2.0: how to train your own model

**DOI:** 10.1038/s41592-022-01663-4

**Published:** 2022-11-07

**Authors:** Marius Pachitariu, Carsen Stringer

**Affiliations:** Howard Hughes Medical Institute (HHMI) Janelia Research Campus, Ashburn, VA USA

**Keywords:** Image processing, Computational platforms and environments

## Abstract

Pretrained neural network models for biological segmentation can provide good out-of-the-box results for many image types. However, such models do not allow users to adapt the segmentation style to their specific needs and can perform suboptimally for test images that are very different from the training images. Here we introduce Cellpose 2.0, a new package that includes an ensemble of diverse pretrained models as well as a human-in-the-loop pipeline for rapid prototyping of new custom models. We show that models pretrained on the Cellpose dataset can be fine-tuned with only 500–1,000 user-annotated regions of interest (ROI) to perform nearly as well as models trained on entire datasets with up to 200,000 ROI. A human-in-the-loop approach further reduced the required user annotation to 100–200 ROI, while maintaining high-quality segmentations. We provide software tools such as an annotation graphical user interface, a model zoo and a human-in-the-loop pipeline to facilitate the adoption of Cellpose 2.0.

## Abstract (web summary)

Cellpose 2.0 improves cell segmentation by offering pretrained models that can be fine-tuned using a human-in-the-loop training pipeline and fewer than 1,000 user-annotated regions of interest.

## Main

Biological images of cells are highly diverse due to the combinatorial options provided by various microscopy techniques, tissue types, cell lines, fluorescence labeling and so on^1–4^. The available options for image acquisition continue to diversify as advances in biology and microscopy allow for monitoring a larger diversity of cells and signals. This diversity of methods poses a grand challenge to automated segmentation approaches, which have traditionally been developed for specific applications, and fail when applied to new types of data.

High-performance segmentation methods now exist for several applications^5–9^. These algorithms typically rely on large training datasets of human-labeled images and neural network-based models trained to reproduce these annotations. Such models draw heavy inspiration from the machine vision literature of the last 10 years, which is dominated by neural networks. However, neural networks struggle to generalize to out-of-distribution data, that is new images that look fundamentally different from anything seen during training. To mitigate this problem, machine vision researchers assemble diverse training datasets, for example by scraping images from the internet or adding perturbations^10,11^. Computational biologists have tried to replicate this approach by constructing training datasets that were either diverse (Cellpose) or large (TissueNet, LiveCell). Yet even models trained on these datasets can fail on new categories of images (for example, the Cellpose model on TissueNet or LiveCell data: Fig. 3a,c).

Thus, a challenge arises: how can we ensure accurate and adaptable segmentation methods for new biological image types? Recent studies have suggested new architectures, new training protocols and image simulation methods for attaining high-performance segmentation with limited training data^12–15^. An alternative approach is provided by interactive machine learning methods. For example, methods such as Ilastik allow users to both annotate their data and train models on their own annotations^16^. Another class of interactive approaches known as ‘human-in-the-loop’ start with a small amount of user-segmented data to train an initial, imperfect model. The imperfect model is applied to other images, and the results are corrected by the user. This is the strategy used to annotate the TissueNet dataset, which in total took two human years of crowdsourced work for 14 image categories^6,17^. The annotation/retraining process can also be repeated in a loop until the entire dataset has been segmented. This approach has been demonstrated for simple ROI such as nuclei and round cells, which allow for weak annotations such as clicks and squiggles^18,19^, but not for cells with complex morphologies that require full cytoplasmic segmentation. For example, using an iterative approach^19^, a 3D dataset of nuclei was segmented in approximately one month. It is not clear whether the human-in-the-loop approach can be accelerated further, and whether it can in fact achieve human levels of accuracy on cellular images.

Here we developed algorithmic and software tools for adapting neural network segmentation models to new image categories with very little new training data. We demonstrate that this approach is: (1) necessary, because annotation styles can vary dramatically between different annotators; (2) efficient, because it only requires a user to segment 500–1,000 ROI offline or 100–200 ROI with a human-in-the-loop approach and (3) effective, because models created this way have similar accuracy to human experts. We performed these analyses on two large-scale datasets released recently^6,7^ and we used Cellpose, a generalist model for cellular segmentation^5^. We took advantage of these new datasets to develop a model zoo of pretrained models, which can be used as starting points for the human-in-the-loop approach. We also developed a user-friendly pipeline for human-in-the-loop annotation and model retraining. An annotator using our graphical user interface (GUI) was able to generate state-of-the-art models in 1–2 hours per category.

## Results

### Human annotators use diverse segmentation styles

**Fig. 1:**
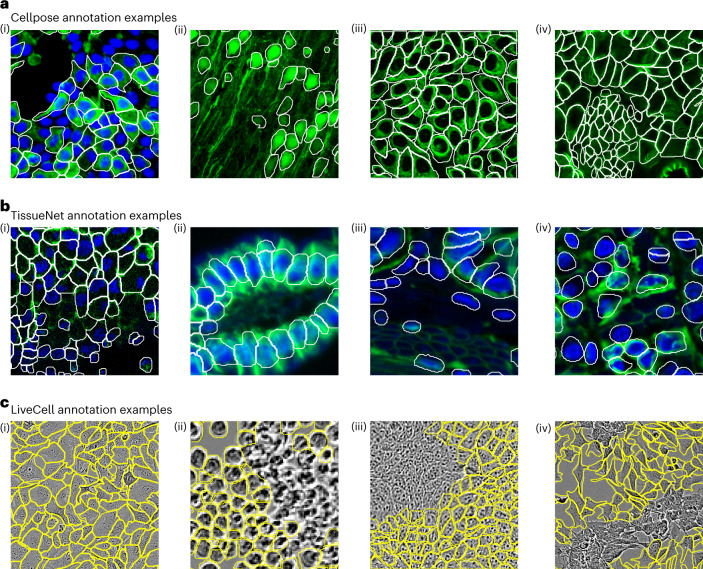
Diverse annotation styles across ground-truth datasets. These are examples of images that the human annotators chose to segment a certain way, where another equally valid segmentation style exists. All these examples were chosen to be representative of large categories of images in their respective datasets. **a**, Annotation examples from the Cellpose dataset. From left to right, these show: (i) nuclei without cytoplasm are not labeled, (ii) diffuse processes are not labeled, (iii) outlines biased toward the outside of cells and (iv) dense areas with unclear boundaries are nonetheless segmented. **b**, Annotation examples from the TissueNet dataset. These illustrate: (i) outlines follow membrane/cytoplasm for some image types, and include nuclei without a green channel label, (ii) outlines do not follow cytoplasm for other image types, (iii) slightly out of focus cells are not segmented and (iv) outlines drawn just around nucleus for some image types. **c**, Annotation examples from the LiveCell dataset. These illustrate: (i) dense labeling for some image types, (ii) no labeling in dense areas for other image types, (iii) same as (ii) and (iv) no labeling in some image areas for unknown reasons.

The original Cellpose is a generalist model that can segment a wide variety of cellular images^5^. We gradually added more data to this model based on user contributions, and we wanted to also add data from the TissueNet and LiveCell datasets^6,7^. However, we noticed that many of the annotation styles in the new datasets were conflicting with the original Cellpose segmentation style. For example, nuclei were not segmented in the Cellpose dataset if they were missing a cytoplasm or membrane label (Fig. 1a(i)), but they were always labeled in the TissueNet dataset (Fig. 1b(i),(iii)). Processes that were diffuse were not segmented in the Cellpose dataset (Fig. 1a(ii)) but they were always segmented in the LiveCell dataset (Fig. 1c(iv)). The outlines in the Cellpose dataset were drawn to include the entire cytoplasm of each cell, often biased toward the exterior of the cell (Fig. 1a(iii)). Some TissueNet categories also included the entire cytoplasm (Fig. 1b(i)), but others excluded portions of the cytoplasm (Fig. 1b(ii),(iii)) or even focused exclusively on the nucleus (Fig. 1b(iv)). Finally, areas of high density and low confidence were nonetheless given annotations in the Cellpose dataset and in some LiveCell categories (Fig. 1a(iv),c(i)), while they were often not segmented in other LiveCell categories (Fig. 1c(ii)–(iv)).

### Creating a model zoo for Cellpose

These examples of conflicting segmentations were representative of large classes of images from across all three datasets. Given this variation in segmentation styles, we reasoned that a single global model may not perform best on all images. Thus, we decided to create an ensemble of models that a user can select between and evaluate on their own data. This would be similar to the concept of a ‘model zoo’ available for other machine learning tasks^20–22^, and similar to a recent model zoo for biological segmentation^23^.

To synthesize a small ensemble of models, we developed a clustering procedure that groups images together based on their segmentation style (also ref. ^12^). As a marker of the segmentation style, we used the style vectors from the Cellpose model^5,24^. This representation summarizes the style of an image with a ‘style vector’ computed at the most downsampled level of the neural network. The style vector is then broadcast broadly to all further computations, directly affecting the segmentation style of the network. Conventionally, this style vector would be referred to as an ‘image style’; however, in this case the segmentation is strongly correlated with the image type, so the style computed here also contains information about the segmentation.

**Fig. 2:**
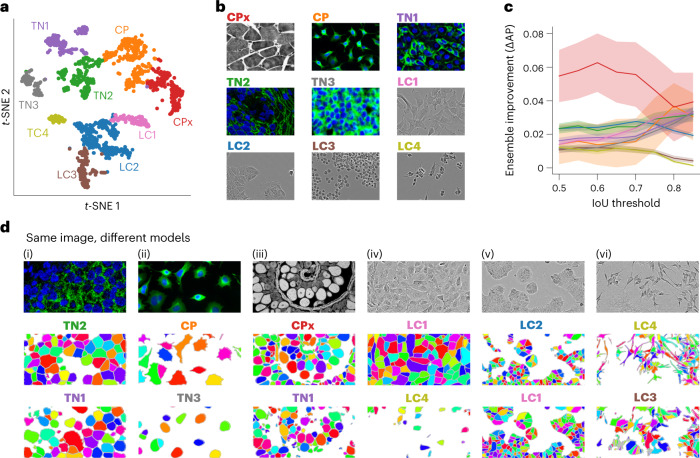
An ensemble of models with different segmentation styles. **a**, *t*-SNE display of the segmentation styles of images from the Cellpose, LiveCell and TissueNet datasets. The style vector computed by the neural network was embedded in two-dimensions using *t*-SNE and clustered into nine groups using the Leiden algorithm. Each color indicated one cluster, with the name chosen based on the most popular image category in the cluster. **b**, Example images from each of the nine clusters corresponding to different segmentation styles. **c**, Improvement of the generalist ensemble model compared to a single generalist model. **d**, Examples of six different images from the test set segmented with two different styles each. Error bars represent the s.e.m. across test images.

We took the style vectors for all images and clustered them into nine different classes using the Leiden algorithm, illustrated on a *t*-SNE (*t*-distributed stochastic neighbor embedding) plot in Fig. 2a (refs. ^25,26^). For each class, we assigned it a name based on the most common image type included in that class. There were four image classes composed mainly of fluorescent cell images (CP, TN1, TN2, TN3), four classes composed mainly of phase-contrast images (LC1, LC2, LC3, LC4) and a ninth class including a wide variety of images (CPx) (Fig. 2b). For each cluster, we trained a separate Cellpose model. At test time, new images were co-clustered with the predetermined segmentation styles and automatically assigned to one of the nine clusters. Then the specific model trained on that class was used to segment the image. The ensemble of models significantly outperformed a single global model (Fig. 2c). All image classes had improvements in the range of 0.01–0.06 for the average precision score, with the largest improvements observed at higher intersection-over-union (IoU) thresholds, and for the most diverse image class (CPx). This suggests that the original Cellpose model may generalize across varying image types, but cannot generalize across different segmentation styles.

Having obtained nine distinct models, we investigated differences in segmentation style by applying multiple models to the same images (Fig. 2d). We saw a variety of effects: the TN1 model drew smaller regions around each nucleus than the TN2 model, which extended the ROI until they touched each other (Fig. 2d(i)); the CP model carefully tracked the precise edges of cells while the TN3 model ignored processes (Fig. 2d(ii)); the CPx model segmented everything that looked like an object, while the TN1 model selectively identified only bright objects, assigning dim objects to the background (Fig. 2d(iii)); the LC1 model overall identified more cells than the LC4 model, which specifically ignored larger ROI (Fig. 2d(iv)); the LC2 model ignored ROI in very dense regions, unlike the LC1 model that segmented everything (Fig. 2d(v)) and the LC4 model tracked and segmented processes over longer distances than the LC3 model (Fig. 2d(vi)) and so on. None of these differences are mistakes. Instead, they are different styles of segmenting the same images, each of which may be preferred by a user depending on circumstances. By making these different models available in Cellpose 2.0, we empower users to select the model that works best for them. Further, we added a ‘suggestion mode’ to automatically select the model that best matches the style of the user image.

**Extended Data Fig. 1:**
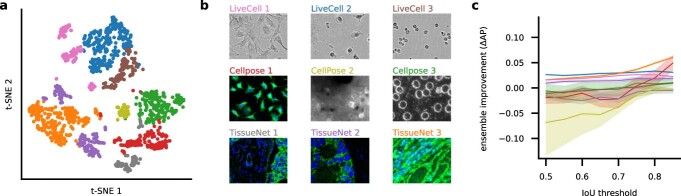
Specialization of Cellpose model without trained style. **a**, t-SNE embedding of segmentation styles for each image, colored according to cluster identity. **b**, Representative example images from each class. **c**, AP improvement of the model ensemble over a single generalist model. Error bars represent the standard error of the mean across test images.

**Extended Data Fig. 2:**
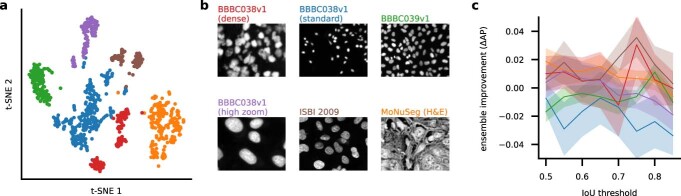
Specialization of the pretrained model for images of nuclei. **a**, t-SNE embedding of segmentation styles for each image, colored according to cluster identity. **b**, Representative example images from each class. **c**, AP improvement of the model ensemble over a single generalist model. Error bars represent the standard error of the mean across test images.

We also find that the specific neural network architecture used in Cellpose may aid in identifying segmentation styles: a network that does not broadcast the style vector to subsequent layers does not show any improvement for the ensemble model over the generalist model (Extended Data Fig. 1). We repeated the style clustering procedure to generate ensembles of models for nuclear segmentation. However, we did not see an improvement for the ensemble of models compared to the generalist model (Extended Data Fig. 2), consistent with the results of ref. ^27^.

### Cellular segmentation without big data

We have seen so far that segmentation styles can vary significantly between different datasets, and that an ensemble of models with different segmentation styles can in fact outperform a single generalist model. However, some users may prefer segmentation styles not available in our training set. In addition, the ensemble method does not address the out-of-distribution problem, that is, the lack of generalization to completely new image types. Therefore, we next investigated whether a user could train a completely custom model with relatively little annotation effort.

For this analysis, we treated the TissueNet and LiveCell datasets as new image categories, and asked how many images from each category are necessary to achieve high performance. We used as baselines the models shared by the TissueNet and LiveCell teams (‘Mesmer’ and ‘LiveCell model’), which were trained on their entire respective datasets. We trained new models based on the Cellpose architecture that were either initialized with random weights (‘from scratch’), or initialized with the pretrained Cellpose weights and trained further from there (also ref. ^14^). The diversity of the Cellpose training set allows the pretrained Cellpose model to generalize well to new images, and provides a good starting set of parameters for further fine-tuning on new image categories. The pretraining approach has been successful for various machine vision problems^28–30^.

The TissueNet dataset contained 13 image categories with at least ten training images each, and the LiveCell dataset contained eight. We trained models on image subsets containing different numbers of training images. To better explore model performance with very limited data, we split the 512 × 512 training images from the TissueNet dataset into quarters. We furthermore trained models on a quarter of a quarter image, and a half of a quarter image. For testing, we used the images originally assigned as test images in each of these datasets.

**Fig. 3:**
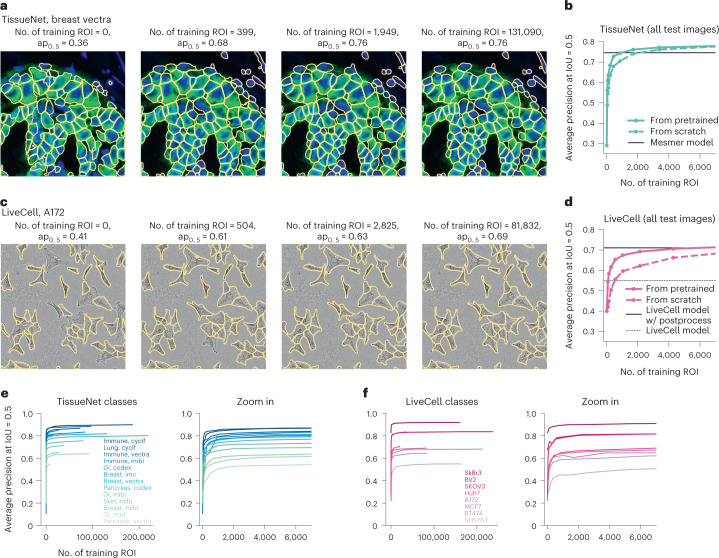
State-of-the-art cellular segmentation does not require big data. **a**, Segmentation of the same test image by models trained with incrementally more images and initialized from the pretrained Cellpose 1.0 model. The image category is breast, vectra from the TissueNet dataset. **b**, Average precision of the models as a function of the number of training masks. Shown is the performance of models initialized from the Cellpose parameters or initialized from scratch. We also show the performance of the Mesmer model, which was trained on the entire TissueNet dataset. **c**,**d**, Same as **a**,**b** for image category A172 from the LiveCell dataset. The LiveCell model is shown as a baseline, with the caveat that this model was trained to report overlapping ROI (Methods). **e**, Left shows the average precision curves for all image categories in the TissueNet dataset. Right shows a zoom-in for less than 3,000 training masks. **f**, Same as **e** for the LiveCell image categories.

Figure 3a shows segmentations of four models on the same image from the test set of the ‘breast vectra’ category of TissueNet. The first model was not trained at all, and illustrates the performance of the pretrained Cellpose model. The second model was initialized with the pretrained Cellpose model, and further trained using four 256 × 256 images from the TissueNet dataset. The third model was trained with 16 images, and the fourth model used all 524 available images. The average precision score for the test image improved dramatically from 0.36 to 0.68 from the first to the second model. Much smaller incremental improvements were achieved for the third and fourth models (0.76 and 0.76). The rapid initial improvement is also seen on average for multiple models trained with different subsets of the data and on all TissueNet categories (Fig. 3b). Furthermore, pretrained Cellpose models improved faster than the models trained from scratch: the pretrained model reaches an average precision of 0.73 at 426 training ROI versus 0.68 average precision for the model trained from scratch. We also noticed that the pretrained Cellpose models outperform the strong Mesmer model starting at 1,000 training ROI, which corresponds to two full training images (512 × 512). This increase in performance happens despite the Mesmer model being trained with up to 200,000 training ROI from each image category, and is likely explained by differences between the architecture of the segmentation models.

**Extended Data Fig. 3:**
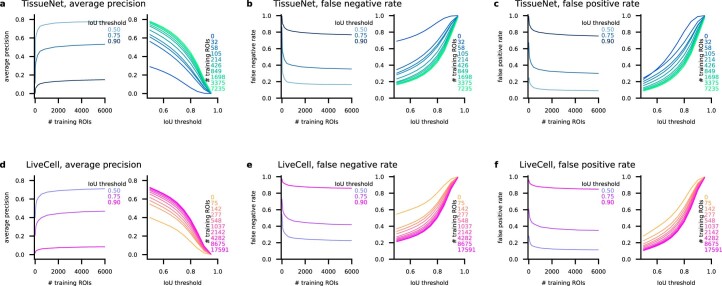
Segmentation performance for different metrics. **a**, Average precision of segmentation on the TissueNet test set for additional IoU thresholds (IoU threshold = 0.5 also shown in Fig. 3b). Performance shown as a function of the number of training ROIs for models initialized from the pretrained Cellpose model. (left) Average precision as a function of training ROIs at three IoU thresholds; (right) average precision as a function of IoU threshold for different numbers of training ROIs. **b**, False negative rates on the TissueNet test set. (left) False negative rates as a function of training ROIs at three IoU thresholds; (right) false negative rates as a function of IoU thresholds for different numbers of training ROIs. **c**, Same as (b) for the false positives rates. **d**-**f** Same as (a-c) for the LiveCell dataset.

We see a similar performance scaling for images from the ‘A172’ category of the LiveCell dataset (Fig. 3c,d). Performance improves dramatically with 504 training ROI (equivalent to two training images), and then improves much more slowly until it reaches the maximum at 81,832 ROI. The Cellpose models also outperform the LiveCell model released with the LiveCell dataset^31^. Finally, we see similar performance scaling across all image categories from both datasets (Fig. 3e,f), and using different quality metrics (Extended Data Fig. 3). We conclude that 500–1,000 training ROI from each image category are sufficient for near-maximal segmentation accuracy in the TissueNet and LiveCell datasets.

**Extended Data Fig. 4:**
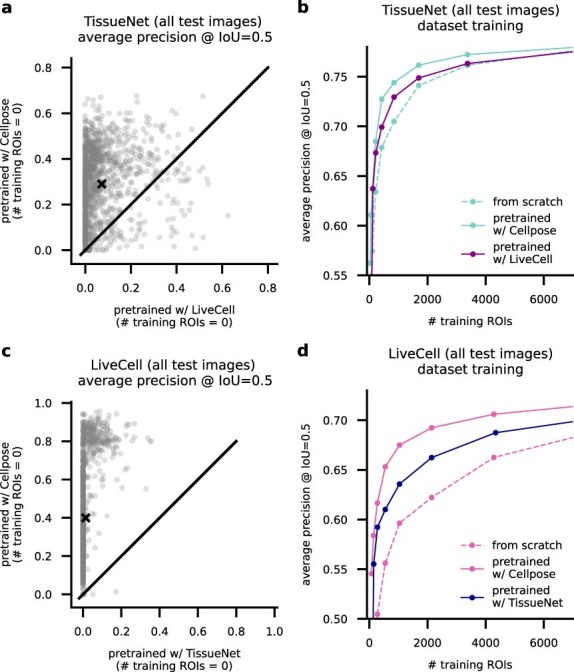
Models pretrained on Cellpose dataset outperform models pretrained on other datasets. **a**, Average precision at IoU threshold 0.5 on the TissueNet test set for the Cellpose model pretrained on the LiveCell dataset versus pretrained on the Cellpose dataset. **b**, Average precision on the TissueNet test set as a function of the number of training ROIs, for models 1) trained from scratch, 2) pretrained with the Cellpose dataset (same as Fig. 3b), or 3) pretrained with the LiveCell dataset. **c**, Average precision on the LiveCell test set for the Cellpose model pretrained on the TissueNet dataset versus pretrained on the Cellpose dataset. **d**, Average precision at IoU threshold on the LiveCell test set as a function of the number of training ROIs from the LiveCell dataset, for models 1) trained from scratch, 2) pretrained with the Cellpose dataset (same as Fig. 3d), or 3) pretrained with the TissueNet dataset.

We next tested whether it matters which dataset Cellpose was pretrained on. We find that pretraining on the Cellpose dataset provided an advantage over pretraining on the TissueNet and LiveCell datasets (Extended Data Fig. 4). The Cellpose dataset is smaller but more diverse than the TissueNet and LiveCell datasets. These results thus indicate that diversity matters more than size for pretraining segmentation models.

### Fast modeling with a human-in-the-loop approach

**Fig. 4:**
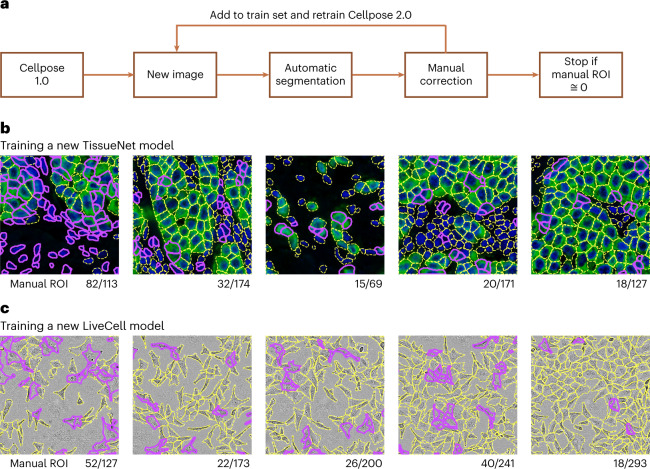
A human-in-the-loop approach for training specialized Cellpose models. **a**, Schematic of human-in-the-loop procedure. This workflow is available in the Cellpose 2.0 GUI. **b**, A new TissueNet model on the breast, vectra category was built by sequentially annotating the five training images shown. After each image, the Cellpose model was retrained using all images annotated so far and initialized with the Cellpose parameters. On each new image, the latest model was applied and the human curator only added the ROI that were missed or incorrectly segmented by the automated method. The yellow outlines correspond to cells correctly identified by the model, and the purple outlines correspond to the new cells added by the human annotator. **c**, Same as **b** for training a LiveCell model on the A172 category.

**Extended Data Fig. 5:**
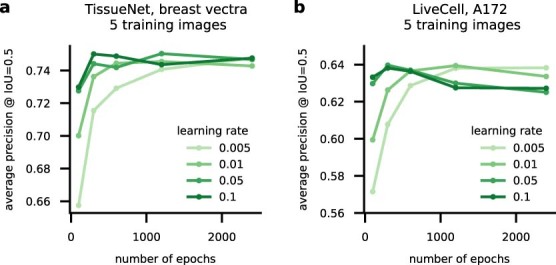
Test set performance as a function of learning rate and training epochs. **a**, Average precision for models trained on the Tissuenet dataset, using 5 training images. **b**, Same as (a) for the Livecell dataset.

We have shown in the previous section that good models can be obtained with relatively few training images when starting from the Cellpose pretrained model. We reasoned that annotation times can be reduced further if we used a ‘human-in-the-loop’ approach^6,19,32^. We therefore designed an easy-to-use, interactive platform for image annotation and iterative model retraining. The user begins by running one of the pretrained Cellpose models (for example, Cellpose 1.0: Fig. 4a). Using the GUI, the user can correct the mistakes of the model on a single image and draw any ROI that were missed or segmented incorrectly. Using this image with ground-truth annotation, a new Cellpose model can be trained and applied to a second image from the user’s dataset. The user then proceeds to correct the segmentations for the new image, and then again retrains the Cellpose model with both annotated images and so on. The user stops the iterative process when they are satisfied with the accuracy of the segmentation. In practice, we found that 3–5 images were generally sufficient for good performance. Further, we found that large learning rates performed well when retraining Cellpose on a small set of images (Extended Data Fig. 5). Therefore, we used a default of 100 training epochs for model retraining, which results in run times that are very short (<1 minute on a graphical processing unit (GPU)).

To assess the performance of this platform, we trained multiple models with various human-in-the-loop and offline annotation strategies. Critically, we used the same human to train all models, to ensure that the same segmentation style is used for all models. We illustrate two example timelines of the human annotation process (Fig. 4bc). For the TissueNet category, the human annotator observed that many cells were correctly segmented by the pretrained Cellpose model, but nuclei without cytoplasm were always ignored, which is likely due to the segmentation style used in the original Cellpose dataset (Fig. 1a(i)). Hence, 82 new ROI were added and the model was retrained. On the next image, only 32 new ROI had to be manually added, which continued to decrease on the third, fourth and fifth images. Qualitatively, the human annotator observed that the model’s mistakes were becoming more subjective, and were often due to uncertain cues in the image. Nonetheless, the annotator continued to impose their own annotation style, to ensure that the final model captured a unique, consistent style at test time. A similar process was observed for images from the LiveCell dataset (Fig. 4c), where 52 out of 127 ROI had to be drawn manually on the first image, but only 18 out of 293 ROI had to be drawn on the fifth image.

**Fig. 5:**
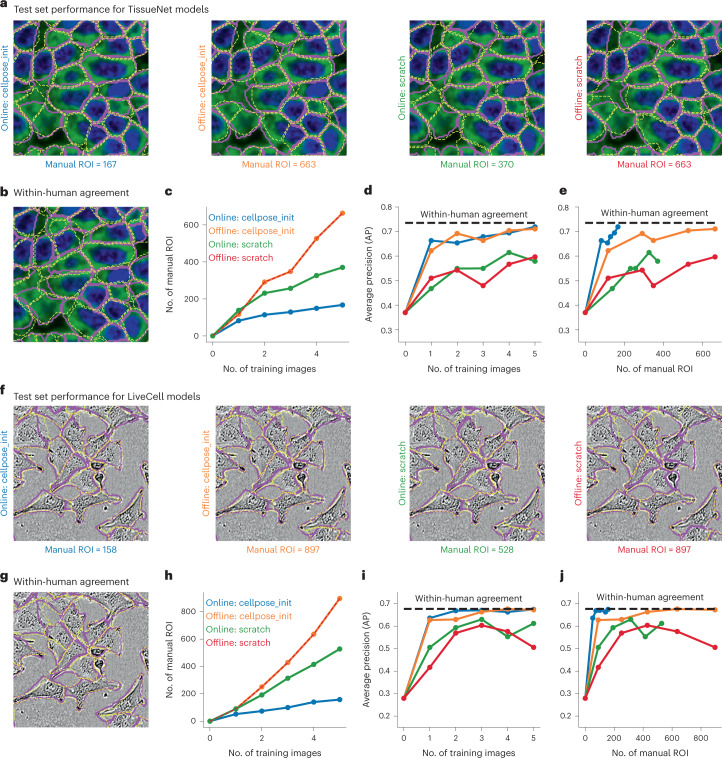
Human-in-the-loop models require minimal human annotation. **a**, Test image segmentations of four models trained on the five TissueNet images from Fig. 4b with different annotation strategies. Annotations were either produced with a human-in-the-loop approach (online), or by independently annotating each image without automated help (offline). The models were either pretrained (cellpose_init) or initialized from scratch. Purple outlines correspond to the ground-truth provided by the same annotator. Yellow outlines correspond to model predictions. **b**, Within-human agreement was measured by having the human annotator segment the same test images twice. For the second annotation, the images were mirrored horizontally and vertically to reduce memory effects. **c**, Total number of manually segmented ROI for each annotation strategy. **d**, Average precision at an IoU of 0.5 as a function of the number of training images. **e**, Average precision curves as a function of the number of manually annotated ROI. **f**–**j**, Same as **a**–**e** for the image category A172 from the LiveCell dataset. All models were trained on the images from Fig. 4b, with the same annotation strategies.

To evaluate the human-in-the-loop models, we further annotated three test images for each of the two image categories (TissueNet and LiveCell). For comparisons, we also performed complete, offline annotations of the same five training images (from Fig. 4b,c), and we ran the human-in-the-loop procedure with models either initialized from scratch or from the pretrained Cellpose model. Thus, we could compare four different models corresponding to all possible combinations of online/offline training and pretrained/scratch initialization (Fig. 5a). As an upper bound on performance, we annotated the test images twice, with the second annotation performed on images that were mirrored vertically and horizontally (Fig. 5b). The average precision between these two annotations can be used as a measure of ‘within-human’ upper bound. Note that the within-human upper bound is by construction higher than any ‘across-human’ upper bound^6^, because it excludes inconsistencies in segmentation styles between different annotators.

The online models in general required fewer manual segmentations than the offline models (Fig. 5c). Furthermore, the online model initialized from Cellpose required many fewer manual ROI than the online model initialized from scratch. Overall, we only needed to annotate 167 total ROI for the online/pretrained model, compared to 663 ROI for a standard offline approach. Performance-wise, models pretrained with the standard Cellpose dataset did much better than models initialized from scratch (Fig. 5c). Of the four models, the online/pretrained model was unique in achieving near-maximal precision with very few manual ROI (Fig. 5e). All of these results were confirmed with a different set of experiments on a LiveCell image category (Fig. 5f–j). In both cases, 100–200 manually segmented ROI were sufficient to achieve near-maximal accuracy and the process only required 1–2 hours of the user’s time.

## Discussion

Here we have shown that state-of-the-art biological segmentation can be achieved with relatively little training data. To show this, we used two existing large-scale datasets of fluorescence tissue images and phase-contrast images, as well as a new human-in-the-loop approach we developed. We are releasing the software tools necessary to run this human-in-the-loop approach as a part of the Cellpose 2.0 package. Finally, we showed that multiple large datasets can be used to generate a zoo of models with different segmentation strategies, which are also immediately available for Cellpose users.

Our conclusions may seem at odds with the general intuition from the computer vision literature, where large amounts of data are necessary to train powerful models^33,34^. The discrepancy may be due to differences of scope between cell segmentation and general computer vision tasks. Deep learning models for general computer vision tasks need to perform well on a large diversity of test images, and therefore require a large diversity of training images. This is not the case for a typical cell segmentation application, where a model only has to work well on a narrow class of images from the same combination of tissue, microscope and/or dye. Thus, a specialized Cellpose 2 model can perform as well as a state-of-the-art model even with relatively little training data.

Our conclusions may also seem at odds with the conclusions of the original papers introducing the large-scale annotated datasets. The TissueNet authors concluded that performance saturates at 10^4^–10^5^ training ROI. The LiveCell authors concluded that segmentation performance continues to increase when adding more training data. The discrepancy with our results may be due to several factors. First, we found that models initialized with Cellpose saturated their performance much more quickly than models trained from scratch. Second, Cellpose as a segmentation model appeared to perform better than both the Mesmer (TissueNet) and LiveCell models, and this in turn may lead to higher efficiency in terms of required training data. Third, we focused on the initial portion of the performance curves where models were trained on only tens to hundreds of ROI, which was below the first few datapoints considered in the TissueNet and LiveCell studies. We even split images into quarters to explore very limited training data scenarios. Fourth, we used a large set of image augmentations to further increase the diversity of the training set images and improve generalizability^5^. Finally, we point out that the LiveCell study used a different average precision score from ours, which additionally requires a confidence score per ROI, while we used the average precision formulation from the Data Science Bowl challenge and other studies^12,27,35^.

Our analysis also showed that there can be large differences in segmentation style between different annotators, even when their instructions are the same. This variability hints at a fundamental aspect of biological segmentation: there are often multiple correct solutions, and a biologist may prefer one segmentation style over another depending on the purpose of their study. Therefore, the variety of biological segmentation styles cannot be captured by a single, universal model.

Future efforts to release large annotated datasets should focus on assembling highly varied images, potentially using algorithms to identify out-of-distribution cell types^36,37^, and should limit the number of training exemplars per image category. We renew our calls for the community to contribute more varied training data, which is now easy to generate with the human-in-the-loop approach from Cellpose 2.0.

## Methods

The Cellpose code library is implemented in Python v.3 (ref. ^38^), using pytorch, numpy, scipy, numba and opencv^20,39–42^. The GUI additionally uses PyQt and pyqtgraph^43,44^. The figures were made using matplotlib and jupyter-notebook^45,46^.

### Models and training

#### Cellpose model

The Cellpose model is described in detail in ref. ^5^. Briefly, Cellpose is a deep neural network with a U-net style architecture and residual blocks^47,48^. Cellpose predicts three outputs: the probability of a pixel being inside a cell (1), the flows of pixels toward the center of a cell in X (2) and Y (3). The flows are then used to construct the cell ROI. The Cellpose default model (‘cyto’) was trained on 540 images of cells and objects with one or two channels (if the image had a nuclear channel). This is the pretrained model used, which we refer to as the ‘Cellpose 1.0’ model.

#### Training

All training was performed with stochastic gradient descent. In offline mode, the models, either from pretrained or from scratch, were trained for 300 epochs with a batch size of eight, a weight decay of 0.0001 and a learning rate of 0.1. The learning rate increased linearly from 0 to 0.1 over the first ten epochs, then decreased by factors of two every five epochs after the 250th epoch. There were a minimum of eight images per epoch, so if fewer than eight images were in the training set then they were randomly sampled with replacement to create a batch of eight images. In online mode, training occurred for only 100 epochs, otherwise the parameters were the same. The learning rate was again increased linearly from 0 to 0.1 over the first ten epochs, but no annealing of the learning rate occurred toward the end of training. We observed slight performance improvements for the models trained from scratch but not from pretrained for 300 epochs of training compared to 100 epochs.

In Fig. 3, we trained on subsets of images in the training set, from 0.25 (a quarter image), 0.5 (a half image), 1, 2 and 4, in powers of 2 up to 2,048 depending on the number of images in the cell class. We trained at each of these subset sizes five times with five different random subsets of images and averaged the performance and the number of ROI used for training across these five networks.

In Extended Data Fig. 4, we trained models from scratch on all of the TissueNet training set or all of LiveCell training set using the same training parameters as above. These models are included in the model zoo as ‘tissuenet’ and ‘livecell’. We then replicated the protocol in Fig. 3 to determine the retrained performance of these models as a function of the number of training ROI.

The generalist and ensemble models in Fig. 2 and Extended Data Figs. 1 and 2 were trained from scratch for 500 epochs with a batch size of eight, a weight decay of 0.00001 and a learning rate of 0.2. The learning rate increased linearly from 0 to 0.2 over the first ten epochs, then decreased by factors of two every ten epochs after the 400th epoch. The model used to compute style vectors in Fig. 2a was trained with images sampled from the Cellpose ‘cyto’ dataset, the TissueNet dataset and the LiveCell dataset, with probabilities 60, 20 and 20%, respectively. The generalist model that was compared to the ensembles (Fig. 2c and Extended Data Fig. 1) was trained with images sampled from the style vector clusters with equal probabilities. The ensemble models were trained using all the training images classified in the cluster with equal probability.

For all training, images with fewer than five ROI were excluded.

#### Style clustering and classification

In Cellpose, we perform global average pooling on the smallest convolutional maps to obtain a representation of the style of the image, a 256-dimensional vector^12,24,49^. For the clustering of style vectors in Fig. 2a and Extended Data Fig. 1a we used all of the Cellpose cyto training data (540 images), 20% of the TissueNet training data (521 images) and 20% of the LiveCell training data (638 images). We then ran the Leiden algorithm on these style vectors with 100 neighbors and resolution 0.45 for Fig. 2 and 0.8 for Extended Data Fig. 1 to create nine clusters of images^25^. For the images in the training set not used for clustering and in the test set, we used a *K*-nearest neighbor classifier with a Euclidean distance metric and five neighbors to get their cluster labels.

For the clustering in Extended Data Fig. 2a we used all of the training images in the Cellpose ‘nuclei’ dataset. We then ran the Leiden algorithm on these style vectors with 50 neighbors and resolution 0.25 to create six clusters of images. For the images in the test set, we used a *K*-nearest neighbor classifier with a Euclidean distance metric and five neighbors to get their cluster labels.

#### Evaluation

For all evaluations, the flow error threshold (quality control step) was set to 0.4. When evaluating models on test images from the same image class (Fig. 3), the diameter was set to the average diameter across images in the training set. For the online/offline comparisons in Figs. 4 and 5 the diameter was set to 18 for all the breast vectra TissueNet images and 34 for all the A172 LiveCell images, which was their approximate average diameter in the training set. When evaluating the ensemble versus generalist model performance (Fig. 2 and Extended Data Fig. 1), the diameter was set to the diameter of the given test image for all models, so that we can rule out error variability due to imperfect estimation of object sizes.

### Model comparisons

We compared the performance of the Cellpose models to the Mesmer model trained on TissueNet^6^ and the anchor-free model trained on LiveCell^7,31^.

#### Mesmer model

We used the Mesmer-Application.ipynb notebook provided in the DeepCell-tf github repository to run the model on the provided test images with image_mpp=0.5 and compartment="whole-cell"^6,50^.

#### LiveCell model

We used the pretrained LiveCell anchor-free model provided by the authors to run the model on the provided test images^31,51^. The ROI returned by the algorithm could have overlaps, and therefore we removed the overlaps as described in the LiveCell Dataset section.

The LiveCell model returned a confidence score for each ROI. We postprocessed the ROI returned by the model by removing ROI with a confidence score below 0.45 (Fig. 3d). We then removed any overlapping ROI as described in the LiveCell Dataset section.

#### Quantification of segmentation quality

We quantified the predictions of the algorithms by matching each predicted mask to the ground-truth mask that is most similar, as defined by the IoU metric. Then we evaluated the predictions at various levels of IoU; at a lower IoU, fewer pixels in a predicted mask have to match a corresponding ground-truth mask for a match to be considered valid. The valid matches define the true positives, TP, the ROI with no valid matches are false positives, FP, and the ground-truth ROI, which have no valid match are false negatives, FN. Using these values, we computed the standard average precision metric (AP) for each image:

$$\begin{array}{r}{\mathrm{AP}}=\frac{{\mathrm{TP}}}{{\mathrm{TP}}+{\mathrm{FP}}+{\mathrm{FN}}}.\end{array}$$

The average precision reported is averaged over the average precision for each image in the test set.

#### Human-in-the-loop method

We used an entry-level GPU (Nvidia RTX 2070) for the human-in-the-loop experiments. Run times were relatively short (<1 min) compared to the time it takes to do the manual correction of the ROI. We expect similar run time performance for other GPUs and we expect that retraining times will vary relatively little with the type of GPU used because our batch sizes are small (eight). It is possible, although not desirable, to run the human-in-the-loop process on the CPU, where retraining times of at least several minutes should be expected.

### Datasets

#### TissueNet

The TissueNet dataset consists of 2,601 training and 1,249 test images of six different tissue types collected using fluorescent microscopy on six different platforms, and each image has manual segmentations of the cells and the nuclei (https://datasets.deepcell.org/)^6^. We only used the cellular segmentations in this study. We excluded the ‘lung mibi’ type from Fig. 3 because it only contained one training image and four test images. We thus used the other 13 types: pancreas codex, immune cycif, gi mibi, lung cycif, gi codex, breast vectra, gi mxif, skin mibi, breast mibi, immune vectra, breast imc, immune mibi and pancreas vectra. The training images are 512 × 512 pixels. To enable subsets consisting of fewer ROI in Fig. 3, we divided each training image into four parts and used those in the training protocol.

#### LiveCell

The LiveCell dataset consists of 3,188 training and 1,516 test images of eight different cell lines collected using phase-contrast microscopy, and each image has manual segmentations of the cells (https://sartorius-research.github.io/LIVECell/)^7^. The eight cell lines were MCF7, SkBr3, SHSY5Y, BT474, A172, BV2, Huh7 and SKOV3. The images were segmented with overlaps allowed across ROI. The Cellpose model cannot predict overlapping ROI, therefore the overlapping pixels were reassigned to the mask with the closest centroid. ROI with more than 75% of their pixels overlapping with another ROI were removed. These nonoverlapping ROI were used to train Cellpose and benchmark the results.

For visualization of the LiveCell images in Figs. 3–5, we increased the contrast of the edges in the images by subtracting and dividing by a smoothed version of the image (Gaussian kernel of width 30 pixels).

#### Cellpose cyto dataset

This dataset was described in detail in ref. ^5^. Briefly, this dataset consisted of 100 fluorescent images of cultured neurons with cytoplasmic and nuclear stains obtained from the CellImageLibrary^52^; 216 images with fluorescent cytoplasmic markers from BBBC020 (ref. ^53^), BBBC007v1 (ref. ^54^), mouse cortical and hippocampal cells expressing GCaMP6 using a two-photon microscope and ten images from confocal imaging of mouse cortical neurons with cytoplasmic and nuclear markers, and Google image searches; 50 images taken with standard brightfield microscopy from OMERO^55^ and Google image searches; 58 images where the cell membrane was fluorescently labeled from ref. ^56^ and Google image searches; 86 images from microscopy samples that were either not cells or cells with atypical appearance from Google image searches and 98 nonmicroscopy images of repeating objects from Google image searches.

#### Cellpose nucleus dataset

This dataset was described in detail in ref. ^5^. Briefly this dataset consisted of images from BBBC038v1 (refs. ^27,57^), BBBC039v1 (ref. ^27^), MoNuSeg (ref. ^58^) and ISBI 2009 (ref. ^59^).

### Reporting summary

Further information on research design is available in the Nature Research Reporting Summary linked to this article.

## Online content

Any methods, additional references, Nature Research reporting summaries, source data, extended data, supplementary information, acknowledgements, peer review information; details of author contributions and competing interests; and statements of data and code availability are available at 10.1038/s41592-022-01663-4.

## Supplementary information

Reporting Summary

Supplementary Video 1 Human-in-the-loop annotation and training procedure in the Cellpose GUI with the TissueNet dataset (2× speed).

## Data Availability

No new data were generated in this study because we used publicly available datasets: TissueNet https://datasets.deepcell.org/, LiveCell https://sartorius-research.github.io/LIVECell/ and Cellpose https://www.cellpose.org/dataset. We share a small set of TissueNet images annotated during human-in-the-loop experiments here: https://figshare.com/articles/dataset/Human-in-the-loop_labelled_TissueNet_data_Cellpose_2_0_/20510016.

## Code availability

Cellpose 2.0 was used to perform all analyses in the paper, the code and GUI are available at https://www.github.com/mouseland/cellpose. Scripts for recreating the analyses in Figs. 2 and 3 are available at https://github.com/MouseLand/cellpose/tree/main/paper/2.0. All online analyses were performed using the Cellpose 2.0 GUI. Please see instructions for human-in-the-loop here: https://cellpose.readthedocs.io/en/latest/gui.html#training-your-own-cellpose-model. The Cellpose GUI saves _seg.npy files that contain the ROI found by the algorithm, or the user can save the masks as a tiff in the file menu. An example notebook for training Cellpose 2.0 in the cloud is available at https://colab.research.google.com/github/MouseLand/cellpose/blob/main/notebooks/run_cellpose_2.ipynb.
